# Massive Abdominal Distension in a Five-Year-Old Child: Management of a Giant Mesenteric Cyst

**DOI:** 10.7759/cureus.19598

**Published:** 2021-11-15

**Authors:** Anurag Kumar, Deepak Kumar, Shreekant Bharti, Anil Kumar, Bankim Das

**Affiliations:** 1 Trauma and Emergency, All India Institute of Medical Sciences, Patna, IND; 2 General Surgery, All India Institute of Medical Sciences, Patna, IND; 3 Pathology/Laboratory Medicine, All India Institute of Medical Sciences, Patna, IND; 4 Trauma Surgery, All India Institute of Medical Sciences, Patna, IND; 5 Transfusion Medicine, All India Institute of Medical Sciences, Patna, IND

**Keywords:** abdominal cyst, child, excision, giant cyst, cyst, mesenteric mass

## Abstract

Mesenteric cyst is a rare entity with a very low incidence. The majority of the cases are incidental. Despite several theories, its etiology remains unknown. Some cases present with non-specific symptoms such as pain abdomen, swelling, and abdominal mass. It may rarely get complicated due to hemorrhage, torsion, or rupture of the cyst. Large mesenteric cysts are quite uncommon. However, these cysts seldom grow to produce clinical symptoms arising from compression of adjoining structures, such as vomiting, constipation due to intestinal obstruction, or dyspnoea due to compression of the diaphragm. Despite several theories, its etiology remains unknown. Diagnosis can be achieved with the help of radiological examinations such as ultrasonography (USG), contrast-enhanced computed tomography (CECT), magnetic resonance imaging (MRI), and confirmed by histopathological examination. We report a case of a giant mesenteric cyst in a five-year-old girl.

## Introduction

Mesenteric cysts are rare abdominal tumors with a reported incidence varying in the literature between one in 100,000 and 250,000 [1,2]. The mean age of occurrence is 25 years and can arise anywhere in small bowel mesentery (60%), large bowel mesentery (24%), and retroperitoneum (16%) [3]. Diagnosis requires a high degree of clinical suspicion during the examination, and it can be adequately narrowed down with the help of radiological examinations, such as CT, MRI, etc.

Treatment requires complete surgical removal either by open or laparoscopy method. Complete surgical excision is important to avoid a risk of recurrence [4]. We present a case of a five-year-old girl with a giant mesenteric cyst occupying almost her entire abdomen and had pushed the entire gut superiorly.

## Case presentation

**Figure 1:**
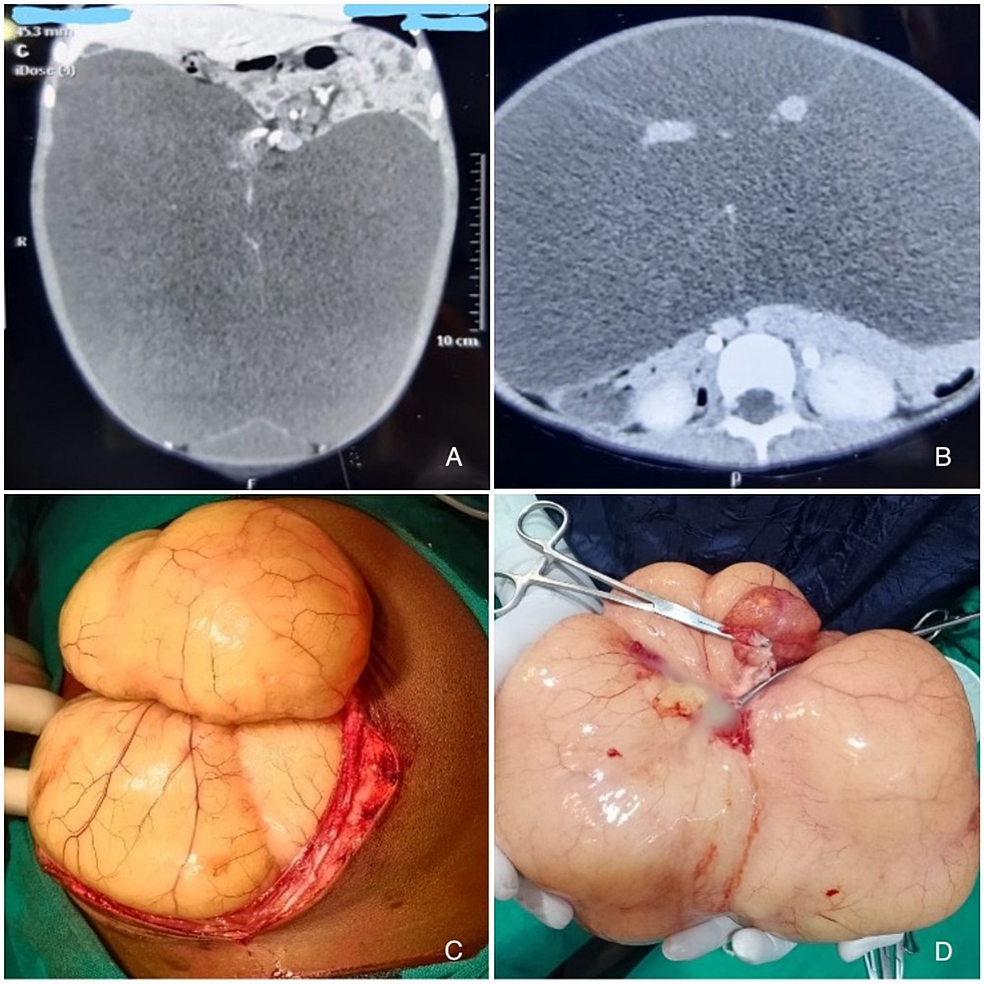
CECT and intra-operative images of mesenteric cyst. (A and B) Contrast-enhanced computed tomography images of the abdomen showed a large fluid-filled cyst occupying the entire abdominal cavity. The bowel loops were pushed aside. (C) Intraoperative bulging out of the cyst from the abdominal incision. (D) Excised cyst with a loop of the attached duodenal segment. CECT: Contrast-enhanced computed tomography

A five-year-old female child arrived at the emergency department with a hugely distended abdomen. She had a history of abdominal distension for the last two years. The patient was not able to squat because of her massively distended abdomen. She complained of respiratory distress and repeated vomiting on taking meals. On examination, the abdominal swelling was soft in consistency with palpable fluid wave. However, there was no shifting dullness appreciated. Her routine investigations were insignificant except for a slightly raised leucocyte count. Contrast-enhanced computed tomography (CECT) of the whole abdomen showed a large cyst occupying the entire abdominal cavity. The small gut was pushed to the left upper quadrant of the abdomen (Figures 1A, 1B).

Because of the large size, a laparoscopic approach to remove the cyst was deferred. The abdomen was explored by a midline incision under general anesthesia. The cyst bulged out of the incision with the adjoining involved gut depicting the highly increased intra-abdominal pressure (Figure 1C). The cyst was involving around 51 cm of ileum, approximately 76 cm proximal to the ileocecal junction. It was not possible to dissect out the cyst from mesenteries of ileum so resection of the small gut with involved mesentery was done followed by end-to-end anastomosis. The abdomen was closed in layers with an intrapelvic drain. The specimen was sent for histopathological examination. A large multilocular cyst weighing 3.9 kg showed a bosselated, yellowish glistening outer surface (Figure 1D). On cutting open, around 3 L of straw to white-colored fluid came out. The inner wall of the cyst was smooth. On microscopic examination, the cyst wall was composed of fibrous tissue and was lined by bland appearing flattened endothelium. Focal mild lymphocyte infiltrate was noted within the fibrous wall. No microscopic features of invasion were noted anywhere. A histopathological diagnosis of a benign chylolymphatic cyst was made.

## Discussion

The first report of a mesenteric cyst was an incidental autopsy finding dates to 1507 found by Benevenni, Italy. Rokitansky described the features of a mesenteric cyst which was published in a handbook of pathology [1]. However, the first successful surgery was performed by Tillaux in 1880. A mesenteric cyst is defined as a cyst located in the mesentery with a recognizable lining of endothelial or mesothelial cells. It can occur anywhere in the mesentery from the duodenum to rectum. Around 60% of cysts occur in the small-bowel mesentery, 24% in large bowel mesentery, and 14.5% in retroperitoneum [2]. The mesenteric cyst can be simple, unilocular, or multilocular and they may contain hemorrhagic, chylous, or infected fluid.

The most accepted classification was given by Beahrs et al. in 1955, in which cysts were grouped into four types such as developmental, traumatic, infectious, and neoplastic. They can range in size from a few millimeters to a few centimeters in diameter. But in some cases, a massively enlarged cyst may mimic ascites, as in our case [3]. Though the exact etiology is not clear, failure of communication between lymph nodes and the lymphatic or venous system or, obstruction as a result of trauma, infection, or neoplasm can contribute to pathogenesis [4]. The mesenteric cyst can occur at any age. But more than 33% of cases occur in children less than 15 years of age [5]. The clinical presentation can be quite varied. Patients may be completely asymptomatic or may present with symptoms of acute abdomen. It depends on the location of the cyst, size, and complications. The complications are rare but include intestinal obstruction, volvulus, shock, peritonitis, hemorrhage, etc.

Assessment requires complete medical history and clinical examination and confirmation by appropriate radio-pathology evaluation. Ultrasound and CT scan easily detect the size, location, thickness of the wall, fluid or solid component, and septation in the cyst. CECT is very much helpful as it was in our case. Treatment of choice is complete surgical excision of the cyst [6]. The surgical approach depends upon the symptoms and present complications. Marsupialization or aspiration of the cyst is not recommended as it is associated with a high rate of recurrence and infection. Localized resection of the bowel or surrounding structure may be required for complete surgical removal of the cyst. The prognosis of a completely excised mesenteric cyst is generally good owing to its benign nature and low recurrence rates. Recurrences, if at all, occur usually within a year. Hence, a short-term follow-up with abdominal ultrasonography suffices.

## Conclusions

A mesenteric cyst should be included in the differential diagnosis of any abdominal mass or distension besides common differential diagnoses. The larger cysts require a high degree of clinical suspicion, as they may fail to produce the relevant clinical signs. Assessment should involve a proper medical history, clinical examination coupled with complete radiological and pathological evaluation. The surgical approach depends upon the size, symptoms, and present complications. A judicious selection of the surgical approach is valuable for a better therapeutic outcome.
